# Inflammation-Related Molecules at the Maternal–Fetal Interface during Pregnancy and in Pathologically Altered Endometrium

**DOI:** 10.3390/cimb44090260

**Published:** 2022-08-23

**Authors:** Wlodzimierz Sieg, Jolanta Kiewisz, Amira Podolak, Grzegorz Jakiel, Izabela Woclawek-Potocka, Jakub Lukaszuk, Krzysztof Lukaszuk

**Affiliations:** 1 Invicta Research and Development Center, 81-740 Sopot, Poland; 2 Department of Human Histology and Embryology, Medical Faculty, Collegium Medicum, University of Warmia and Mazury in Olsztyn, 10-561 Olsztyn, Poland; 3 Department of Obstetrics and Gynecology Nursing, Medical University of Gdansk, 80-210 Gdansk, Poland; 4 The Center of Postgraduate Medical Education, 1st Department of Obstetrics and Gynecology, University of Gdansk, 01-004 Warsaw, Poland; 5 Department of Gamete and Embryo Biology, Institute of Animal Reproduction and Food Research, Polish Academy of Sciences, 10-748 Olsztyn, Poland

**Keywords:** implantation, inflammatory processes, inflammation, endometrium, reproductive immunology, pregnancy, infertility, endometriosis

## Abstract

The blastocyst expresses paternally derived alloantigens and induces inflammation during implantation. However, it is necessary for the onset of pregnancy. An abnormal response might result in a pathological course of pregnancy or pregnancy failure. On the other hand, a state of maternal immune tolerance is necessary to ensure the normal development of pregnancy by suppressing inflammatory processes. This article discusses recognized mechanisms and the significance of inflammatory processes for embryo implantation and pregnancy establishment. We would also like to present disorders involving excessive inflammatory response and their influence on events occurring during embryo implantation. The chain of correlation between the processes responsible for embryo implantation and the subsequent physiological course of pregnancy is complicated. Many of those interrelationships are still yet to be discovered. Undoubtedly, their recognition will give hope to infertile couples for the emergence of new treatments that will increase the chance of giving birth to a healthy child.

## 1. Introduction

The union of the sperm nucleus with the ovum nucleus causes the fusion of the hereditary genetic material in the fertilization process. In physiological conditions, it occurs in the ampulla of the fallopian tube. In humans, after 6–7 days and a series of cell divisions, which result in two-, four-, and eight- cell embryo development, we observe the formation of the cavitated blastocyst. The fluid-filled blastocyst is formed with the trophoblast cells and inner cell mass (embryoblast) under the zona pellucida.

The adherence of the embryo to the inner surface of the uterine wall (endometrium) is called implantation. It is an extraordinarily complex process. It is preceded by the hatching of the blastocyst and divided into apposition, epithelial adhesion, and blastocyst invasion in the endometrial stroma. The invading trophoblast differentiates: syncytiotrophoblast (the outer layer) is in contact with the maternal blood; cytotrophoblast (the inner layer), forming a cytotrophoblastic shell, reduces in time to create the placental membrane. Maternal-derived uterine epithelium (decidua) and fetal-derived placenta form the maternal–fetal interface.

The body’s immune system is educated and programmed to recognize and respond to foreign structures. The situation is much more complicated at the maternal–fetal interface. The mechanisms functioning there are not only designed to protect against pathogens but also provide a support system (created by cells and cytokines) for the mother to protect the embryo and preserve the pregnancy. This system is further modulated by the fetus, which through cytokines of trophoblast origin activates the mother’s immune response, making it possible to maintain the pregnancy under changing environmental conditions [1]. The uterine microbiome also influences the mother’s immune environment, ensuring proper tissue function and immune adaptation of the mother’s endometrium to accept the embryo [2].

**Figure 1:**
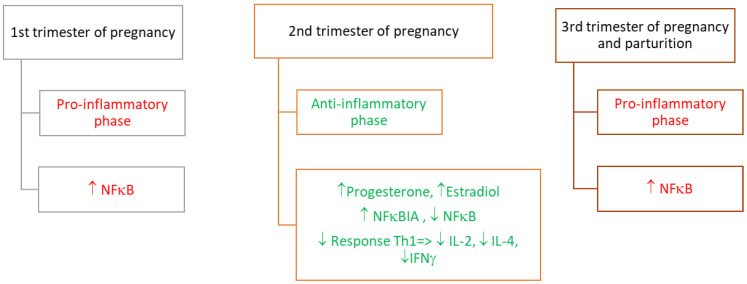
Modulators of pro- and anti-inflammatory stages (the immune phases) during pregnancy. Legend: ↑: increase; ↓: decrease; IL-2: interleukin 2; IL-4: interleukin 4; IFNγ: interferon gamma; NFκB: nuclear factor kappa-light-chain-enhancer of activated B cells; NFκBIA: NFκB inhibitor alpha; Th: T helper cells.

During pregnancy, we can distinguish three distinct stages of the immune response (immune phases) [3] (Figure 1). During the first phase, the inflammatory environment establishes the protective surroundings for an implanting embryo. During the second phase, the uterine surrounding promotes fetal growth, thus the immunological reaction is not so intensive. Proinflammatory environments in the uterus appear once again at the moment of parturition. 

The immune microenvironment at the maternal–fetal interface is determined by the presence of cells of the maternal immune system and the secretion of modulating factors by trophoblast cells. Since the secretory molecules modulate the pro- and anti-inflammatory environment of the uterus during pregnancy, we decided to summarize the current knowledge of acute inflammation and the molecules engaged in this process at the maternal–fetal interface. Particular attention was paid to regulation at the molecular level by the nuclear factor kappa-light-chain-enhancer of activated B cells (NFκB) under physiological conditions. The contributions of prostaglandins—the main regulators of inflammation—were not neglected. Aspects concerning the pathological state of chronic inflammation in the endometrium and its influence on fertility were also considered.

## 2. Inflammation-Related Molecules at the Maternal–Fetal Interface

Before blastocyst invasion, endometrial stromal cells secrete pro-inflammatory cytokines such as tumor necrosis factor α (TNF-α) and interleukin 1β (IL-1β) [4,5] to initiate inflammation in the uterine mucosa. The early detection of these cytokines in the endometrium was demonstrated to be the molecular marker of implantation [5]. Both cytokines interact through a feedback loop: IL-1β is the mediator of the immune and inflammatory responses, and its secretion is induced by TNF-α [6,7]. On the other hand, there is an increase in the number of T- helper lymphocytes (Th1) and the synthesis of pro-inflammatory cytokines (IL-1β, interleukins 6 and 8 (IL-6 and IL-8), TNFα and interferon-gamma (IFNγ)) during embryo implantation in the uterine mucosa. Maternal immune response cells such as natural killer (NK) cells, macrophages, and dendritic cells synthesize pro-inflammatory cytokines [6,7,8,9,10,11,12] (Table 1). However, this does not apply to neutrophils [13]. Neutrophils are the first immune cells recruited at the site of infection and usually amplify the inflammatory signal that attracts other immune cells. The mechanisms that prevent neutrophil infiltration into the endometrium are unclear. One likely cause is the suppression of the cytokine signaling involved in their recruitment [11,14].

### 2.1. Involvement of the Transcription Factor—NFκB in the Inflammatory Response

The accumulation of immune cells at the maternal–fetal interface and the secretion of inflammatory mediators during implantation occur under the control of NFκB. NFκB is a transcription factor involved in the regulation of the expression of genes associated with the onset of inflammation and generation of the immune response. It is also involved in response to heat stress, apoptosis, and tissue repair. Generally, the NFκB signal transduction pathway is modulated by cytoplasmic inhibitory proteins like inhibitor of nuclear factor-kappa B (IkB), interferon regulatory factor 6 (LPS), TNF, IL-1, or oxidative stress [32,33,34]. The level of NFκB increases during implantation and then subsequently decreases, which determines the maintenance of pregnancy. The re-increase of NFκB before delivery promotes the synthesis of prostaglandins (PGs), cytokines, and chemokines and stimulates uterine contractions [35,36].

Studies have demonstrated the action of NFκB factor and estrogen receptor signaling. Activated NFκB signaling initiates and maintains an inflammatory effect at the cellular level [37,38], while estrogens trigger anti-inflammatory responses [39]. This interaction is integrated by IL-1 [14,15].

Estradiol (E2), which co-operates with estrogen receptors and IL-1β, affecting NFκB signaling, acts synergistically to increase the activity of estrogen response elements (ERE) in the DNA of the endometrial epithelial cells. This interaction increases the expression of the pool of genes involved in implantation, including genes coding prostaglandin E synthase, involved in the synthase of PGE2 [40].

**Figure 2:**
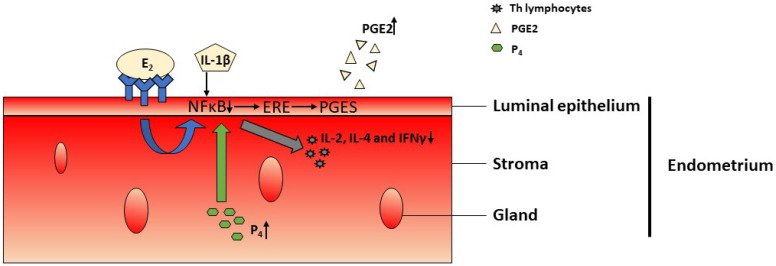
Changes during pregnancy development. Elevated levels of estradiol (E2) together with interleukin 1β (IL-1β) and progesterone (P4) reduce nuclear factor kappa-light-chain-enhancer of activated B cells (NFκB) activation, leading to an increase in the activity of estrogen response elements (ERE) in the DNA of endometrial luminal epithelium. This triggers an increase in the expression of prostaglandin E synthase (PGES) and production of prostaglandin E (PGE2). Reduced NFκB activation causes the inhibition of interleukin 2 (IL-2), interleukin 4 (IL-4) and interferon gamma (IFNγ) production in T lymphocytes. ↑: increase; ↓: decrease.

Another steroid interacting with the NFκB factor is progesterone. The decrease in progesterone synthesis by steroidogenic cells is observed at the beginning of pregnancy. Elevated blood levels of progesterone reduce the expression of its receptors and FκB factor during the peri-implantation period in pigs and rodents [41,42,43,44,45]. Elevated levels of progesterone and estradiol as pregnancy develops (Figure 2.) lead in turn to the increased expression of NFκB inhibitor alpha (NFκBIA) and reduce NFkB activation [35,46]. The inability of NFκB to induce gene expression results in the inhibition of IL-2, IL-4, and IFNγ production by T lymphocytes [47]. These processes are essential for immunosuppression and the maintenance of maternal tolerance of the fetus during pregnancy [48,49]. Pregnancy-specific suppression of NFκB expression plays a role in reducing the production of cytokines by Th1 lymphocytes and maintaining the cytokine profile necessary for pregnancy initiation. On the other hand, NFκB levels in maternal T cells can be regulated not only by maternal steroid hormones or cytokines but also by placental cytokines.

Under the control of NFκB are also the nucleotide-binding oligomerization domain-containing 1 and 2 (NOD1 and NOD2) genes expressed in human fetal membranes and term myometrium at labor. The NOD1 and NOD2 ligands, through NFκB activation, significantly increase proinflammatory and pro-labor mediators in human fetal membranes and myometrium [50]. Shorter gestation was predicted by genome-wide analyses of maternal blood samples when increased NF-κB activity in monocytes was observed [51].

Undoubtedly, the abnormal level of NFκB expression might predispose pregnant women to the pathological course of pregnancy with such consequences as delayed fetal growth, pregnancy-related hypertension, and premature delivery [36,52,53]. Premature or aberrant activation of NFκB factor associated with regulation of pro-inflammatory cytokines action may cause preterm labor [34]. Increased NFκB expression resulting from reduced BCL2 expression was observed in pregnancies complicated by intrauterine growth restriction (IUGR) and preeclampsia [53]. Oxidative stress through increased placental levels of TNFα, COX-2, and thromboxane likely activate placental NF-κB in preeclampsia [52].

### 2.2. Involvement of PGs in the Inflammatory Response

PGs are produced at the time of acute inflammatory reaction. The primary PGs involved in the inflammatory response are prostaglandin E2 (PGE2) and prostaglandin F2α (PGF2α). Studies have shown that in humans, concentrations of PGE2 and PGF2α significantly increase in the fluid obtained from the uterine cavity during the implantation window [54,55]. The PGs are believed to play a significant role in decidualization and trophoblast invasion [39]. PGE2 supports the luteal function of the corpus luteum, essential for embryo development and early implantation. Moreover, it induces the expression of chemokines important for trophoblast apposition and adhesion during implantation [56]. PGE2 has been shown to increase trophoblast adhesion ability via adhesion factors, including integrins [57]. Other studies have demonstrated that increased PGE2 synthesis in endometrial stromal cells contributed to the successful establishment of pregnancy in mammals [58]. Moreover, inhibition of PGE2 synthesis or expression of its receptor disturbed embryo adhesion [54]. Other authors claim that efficient PG synthesis in the endometrium improved implantation rates in patients with repeated implantation failures [59]. Therefore, normalized secretion of PGE2 by endometrial cells is relevant for the receptivity of the endometrium [60] and significantly correlates with the outcome of pregnancy. When the secretion of PGE2 is stable—it improves the effectiveness of embryo implantation. But when it is excessive—the chance for embryo implantation declines [61].

PGF2α can affect a variety of processes, usually acting in opposition to PGE2. PGF2α has been documented to induce: luteolysis [62], proliferation of endometrial epithelial cells [63], and constriction of spiral arterioles as well as contraction of the myometrium [64,65,66]. Moreover, in the endometrial luminal epithelium, PGF2α was found to control sodium and potassium ion transport [67] and induce the expression of endometrial connexins [68,69]. PGF2α causes vasoconstriction and induces hypoxia of endometrial cells. It causes the formation of new blood and lymphatic vessels through a beneficial impact on the production of vascular endothelial growth factor (VEGF) [70] and adrenomedullin [71]. Estrogen was found to stimulate the synthesis of PGF2α while progesterone was found to inhibit it [72,73]. Physiological changes in steroid hormone concentrations during the estrous cycle and pregnancy result in fluctuating levels of PGF2α: the highest levels are observed during implantation and before menstruation [74].

During early pregnancy, PGF2α increases the proliferation of human trophoblast cells [75] and promotes the association of molecules on trophoblast cells to the extracellular matrix protein, specifically fibronectin. Fibronectin expression is increased in the decidua during the first trimester of pregnancy [76,77,78]. Moreover, PGF2α promotes the process of implantation, but its impact can be controlled by the opposing effects of PGE2 [57]. PGF2α causes increased expression of mRNA and subsequent interleukin 6 (IL6) protein production in syncytiotrophoblast cells. The highest expression of IL6 occurs in the middle secretory phase of the menstrual cycle, which corresponds to the time of implantation, which in turn increases the amount of PGF2α in the uterine lumen [79,80]. Moreover, IL6 regulates the activity of matrix metalloproteinases [81] and stimulates the expression of integrins in trophoblast cells and processes such as invasiveness and migration [22,82]. PGF2α also acts indirectly through IL6 and can regulate implantation-related changes and immunological processes such as host defense [83].

On the other hand, pregnancy is associated with an anti-inflammatory condition. The levels of PGF2α metabolite (PGFM) and PGF2α in the decidua were significantly lower in the first trimester of pregnancy, comparable to the secretory phase of the menstrual cycle, when there was earlier elective termination of pregnancy [84]. Increased PGF2α production was shown to cause impaired uterine contractions, resulting in abnormal semen migration, defective transport of fertilized ova, and impaired implantation [85,86]. In women with intramural fibroids, higher levels of PGF2α were found both in the fibroids themselves and in the endometrium, leading to lower pregnancy and implantation rates, even if the fibroids did not distort the uterine cavity [86]. Excessively high levels of PGF2α in decidua may trigger a pregnancy loss cascade and lead to miscarriages [87,88,89].

During implantation, strengthened PGE2 signaling and inhibition of PGF2α signaling within the endometrium were found [61].

The transformation of arachidonic acid to PG precursors is possible due to the action of COX enzymes. Interestingly, COX-2, which is engaged in inflammatory processes, is also involved in the oxidation of endogenous cannabinoid (arachidonoylethanolamide; AEA) [90]. In this way, AEA seems to be capable of modulating PG production [90]. Low levels of serum AEA at the time of implantation were observed in women subjected to in vitro fertilization (IVF) or intra-cytoplasmic sperm injection procedure (ICSI) [91]. The expression of the components of the endocannabinoid system is found in the human placenta at the 30th, 34th, and 40th week of gestation [92]. In the amnion, AEA was found to be responsible for the PGE2 concentration increase [93]. However, it could also cause opposite effects on uterine PGE2 and PGF2α biosynthesis by inhibiting PGE2 production and increasing PGF2α levels [94].

Abnormal PG synthesis was found to be associated with repeated implantation failure in patients undergoing in vitro fertility treatment [59]. Therefore, the measurement of PGs 24 h before the planned embryo transfer allows for the prediction of a favorable outcome [54,55].

## 3. Inflammation-Related Molecules in Pathologically Altered Endometrium

Acute inflammation of the endometrium is essential for successful implantation [95] while chronic inflammation is destructive and can lead to infertility [96,97,98]. Chronic inflammation is caused by endometriosis, chronic endometritis (CE), and hydrosalpinx. Thus, we will briefly characterize these disorders.

Endometriosis is caused by hereditary as well as environmental factors [99]. It affects approximately 190 million women worldwide. The estimated overall prevalence of endometriosis in the population ranges from 0.8% to 6% and is higher among Asian women. The incidence of endometriosis appears to be significantly higher in infertile women than in fertile ones, ranging from 20% to 50%. Differences are also observed depending on the duration of infertility and the age of patients [100,101].

**Figure 3:**
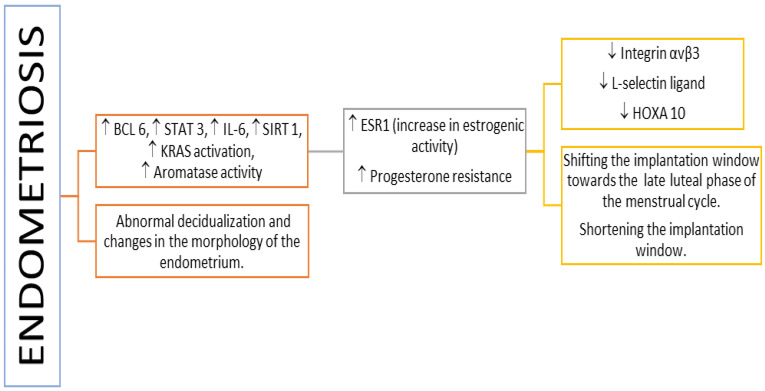
Pathomechanisms impeding the course of implantation in women with endometriosis. Legend: ↑: increase; ↓: decrease; BCL 6: B-cell lymphoma 6; ESR1: estrogen receptor 1; IL-6: interleukin 6; KRAS: gene encoding K-Ras protein with GTPase activity; SIRT 1: Sirtuin 1; STAT3: signal transducer and activator of transcription 3.

Endometriosis is a disease triggered by inflammation induced by estrogens. The local concentration of estrogens and androgens is extremely high compared to peripheral blood concentrations and causes changes in cytokine expression (Figure 3), disrupting the normal function of the endometrium in endometriosis [102,103,104]. Released cytokines involved in immune responses and responsible for inflammation are TNF, IL-1, IL-6, IL-8, IL-10, and TGF-B1 [99]. Other characteristic features of inflammation observed in endometriosis are the infiltration of lymphocytes; synthesis of eicosanoids and metalloproteinases; and atypical changes in the populations of T, B, Treg, and NK lymphocytes. In women with endometriosis, a decrease in gene expression coding for endometrial proteins crucial for proper implantation [96,105], including αVβ3 integrin [105,106], L-selectin ligand [107,108,109], and HOXA10 protein [110,111,112], was observed.

Moreover, many studies have shown abnormal decidualization and changes in the morphology of the endometrium [113,114,115,116,117,118]. The observed changes in the expression of endometrial genes are caused by excessive estrogenic activity [119,120]. In patients with endometriosis, it was demonstrated that the increase in the expression of estrogen receptor (ESR1) occurred during implantation [119,121]. Changes in progesterone receptor (PR) expression and reduction in the effects of progesterone have also been demonstrated [122,123,124,125]. What is more, endometriosis-associated progesterone desensitization contributed to the increased proliferation and survival of cells [126,127] and increased ESR2 levels [121,128]. Insensitivity to progesterone signaling leads to the pro-inflammation condition, as progesterone plays an important role in reducing inflammation in the endometrium [122]. The severity of the inflammatory process and diminished sensitivity of receptors to progesterone differ between women diagnosed with endometriosis [122,129,130]. Increased inflammatory response and reduced progesterone sensitivity are related to a higher risk of implantation failure [129,130,131]. These factors shift the implantation window towards the rest of the menstrual cycle and shorten its duration [130].

Molecular factors involved in cytokine synthesis (as NF-κB factor) and cytokines (as IL-1, IL-2, IL-6, IL-8, IL-33, TNF-α) are potential targets for therapies directed against endometriosis. Extensive laboratory studies utilizing pharmacological inhibitors of NF-κB factor (for example: methyl ester of 2-cyano-3,12-dioxooleana-1,9-dien-28-oicacid, dienogest, thalidomide, genistein, ginsenoside, gossypol), and inhibitors of cytokines (for example: resveratrol, tocilizumab, pyrvinium pamoate, nobiletin, S, R)-3-(4-hydroxyphenyl)-4,5-dihydro-5-isoxazole acetic acid methyl ester [132]) have been conducted. Potential therapies are also being investigated by analyzing the hormone-controlled mechanisms of endometriosis. The most effective solution seems to be lowering estradiol levels by indirectly inhibiting its synthesis using medications such as linzagolix, relugolix, and elagolix [132].

There are treatments for endometriosis that also serve as therapy for endometriosis-associated pain at the same time. Considering their effects on fertility, they have advantages and disadvantages. An improvement in pregnancy rate is offered by the surgical removal of endometriosis lesions or short-term immunotherapy using glucocorticosteroids [99]. Adhesiolysis enhances the chance of spontaneous pregnancy [133]. Therapy with progestins and oestro-progestins influences endometriosis but does not tweak the fertility rate [134]. TNF antagonist treatment seems to be effective but is not recommended for routine usage. The effects of fertility treatment may be worsened by endometriosis immunosuppressive therapy [99].

Another disease characterized by interminable inflammation is chronic endometritis (CE). It is caused by the imbalance between the coexistence of microorganisms on the endometrial surface and the proper function of the immune system manifested by immunocompetent cells in the uterine stroma. Most cases of CE are asymptomatic. Studies have shown that the incidence of CE is 2.8–66.8% in infertile women, 14–67.5% in women with recurrent implantation failure, and 9.3–67.6% in women with recurrent pregnancy loss [135,136].

In approximately 70% of cases, more than one pathogen is responsible for the occurrence of CE. Common bacteria such as *Streptococcus* spp., *Escherichia coli*, *Enterococcus faecalis*, *Klebsiella pneumoniae*, *Staphylococcus* spp., and *Corynebacterium* and *Mycoplasma*/*Ureaplasma* spp. are present in the uterine cavity of CE patients. Their presence was detected by microbial cultures or by PCR tests [137,138,139,140,141,142,143].

CE is diagnosed based on endometrial biopsy and plasma cell presence generated by stimulation of B lymphocytes [144]. The presence of B cells was confirmed in the endometrium throughout the menstrual cycle. They were found mainly in the basal layer and accounted for only a minor percentage (<2%) of all immune cells in the normal endometrium [136,142]. In CE, the B cells number increases significantly in all layers of the endometrium [136,143].

**Figure 4:**
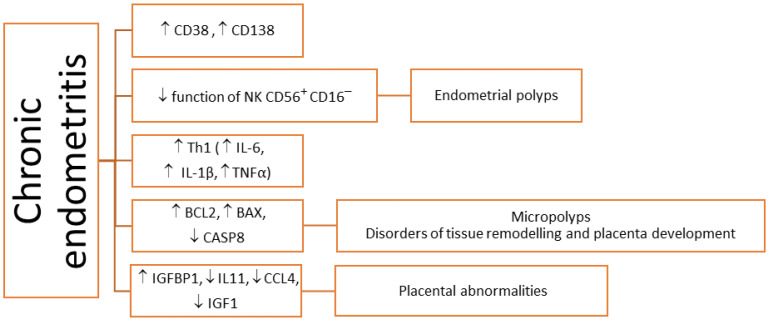
Pathomechanisms impeding the course of implantation in women with chronic endometritis (CE). Legend: ↑: increase; ↓: decrease; BAX: proapoptotic BAX protein; BCL2: anti-apoptotic BCL2 protein; CASP8: cysteine–aspartic acid protease 8; CCL4: CCL4 chemokine; CD38 and CD138: plasma cells CD38 and CD138; IL-1β: interleukin 1β; IL-6: interleukin 6; IL-11: interleukin 11; IGF1: insulin-like growth factor 1; IGFBP1: IGF-binding protein 1; NK CD56 + CD16: decidual NK cells; Th: helper T cells; TNF-α: tumor necrosis factor α.

The immunohistochemical staining of specific surface antigens CD38 and CD138 allows for the detection of plasma cells [144] and the diagnosis of CE with four times greater sensitivity compared to the histopathological evaluation of endometrial tissue sections stained only with hematoxylin and eosin [145]. (Figure 4).

CE can also be diagnosed during hysteroscopic evaluation of the uterine cavity [137,146]. The features indicating the presence of CE are micropolyps, stromal oedema, and focal or diffuse hyperaemia. Hysteroscopic evaluation is more sensitive in the diagnosis of CE than in uterine cavity culture [137]. Some studies found that the proportion of CD56 + CD16– NK cells in the endometrium in the secretory phase was similar in women with unexplained infertility, in CE and control subjects [147,148]; other studies have described significantly higher levels of CD56 + CD16– NK cells in the endometrium of women with CE compared with those without CE [149,150,151,152,153]. Histologically confirmed CE may favor the formation of micropolyps characterized by the accumulation of leukocytes (CD45), macrophages (CD68), plasma cells (CD138), and NK (CD56+) cells, whose activity leads to excess abnormal proliferation of endometrium [149,150]. The distribution of endometrial immunocompetent cells is altered with the menstrual cycle, and the Th1/Th2 balance is Th1-predominant from the menstrual to the proliferative phase, shifting to Th2 predominant from the implantation phase to early pregnancy [151]. The studies revealed that non-CE endometrium showed Th2 predominance in the implantation phase, but CE endometrium showed Th1 predominance [151]. Moreover, increased IL-17 and decreased IL-10 and TGF-β expressions in the endometrium of CE patients were found. This suggests that CE induces a propensity to Th17 over Treg immunity in the endometrium, which consequently leads to poor reproductive outcomes [152].

Women with CE have been found to have increased expression of the insulin-like growth factor-binding protein 1 (IGFBP1) gene in the endometrium, with a simultaneously decreased expression of the insulin-like growth factor 1 (IGF1) genes, IL-11 and CCL4 [153,154]. IGF1 mediates the stimulatory effect of estrogens on the proliferation of endometrial cells, while IGF2 mediates progesterone action during the secretory phase, facilitating embryo implantation and invasion [17,153,155]. Increased secretion of IGFBP1 by the endometrial stromal cell during decidualization counteracts the effect exerted by IGF2, which has a negative impact on embryo implantation. Increased expression of the IGFBP1 gene and decreased expression of the IGF1 gene are responsible for unfavorable conditions for embryo implantation and development. IL-11 is a cytokine with anti-inflammatory properties. IL-11 production is highest during decidualization [156,157]. On the other hand, decreased levels and abnormal IL-11 signaling can disrupt trophoblast invasion [158,159,160].

High expression of the gene encoding transcriptional repressor BCL6 (B-cell lymphoma 6) in the endometrium allows the detection of endometritis associated with endometriosis [161]. Elevated BCL6 and aromatase levels are associated with progesterone resistance and estrogen dominance in women with endometriosis [129]. As a repressor of the genes, BCL6 may be responsible for progesterone resistance by reducing the secretion of progesterone-mediated factors, including the transcription factor that recognizes nucleotide sequence identified in the promoter of a gene encoding chicken ovalbumin upstream promoter-transcription factor 2 (COUP-TFII) [162]. COUP-TFII regulates many genes responsible for the decidualization of the endometrial stromal cells, including those involved in cell adhesion, angiogenesis, and inflammation. COUP-TFII also plays an important role in controlling the expression of inflammatory cytokines [163,164].

During early pregnancy, the trophoblast recruits NK cells and macrophages into the endometrium via chemokines such as CCL4 and stimulates them to produce pro-inflammatory cytokines [153,158,165,166]. Reduced CCL4 activity in women with CE may result in implantation failure or abnormal placental development [153,167,168].

Regarding treatment, personalized oral, systemic antibiotic therapy is considered to be efficient in the therapy of CE [169,170]. Antibiotics such as doxycycline [171] or a combination of levofloxacin and tinidazole [172] are effective in CE treatment. Moreover, they are also considered potentially successful in the improvement of fertility, which was shortly summarized elsewhere [170].

Another disease that can also cause chronic endometritis is hydrosalpinx. Fluid from the fallopian tubes entering the uterine cavity may have a direct embryotoxic effect [173,174]. This fluid contains inflammatory mediators such as cytokines, PGs, mucosa debris and toxins, impairing blood flow through the uterine spiral arteries [175,176,177]. Moreover, hydrosalpinx mechanically disturbs the contact between the embryo and the endometrial surface [173,174]. The effect of hydrosalpinx on the endometrium is chronic endometritis, which negatively affects endometrial receptivity [178,179]. Patients with hydrosalpinx showed a statistically significant increase in the number of many different plasma cells and lymphocytes infiltrating the endometrial stroma, together with the increased expression of IL-2 protein. It is indicative of a generalized inflammation [178,180]. The increased expression of mRNA and NF-κB protein, which promotes inflammatory processes and adversely affects implantation, has also been found [179]. Endometrial HOXA10 implantation factor expression is also reduced in a woman with hydrosalpinx. The salpingectomy procedure regulates HOXA10 expression, improves implantation and reduces early pregnancy loss [181]. Hydrosalpinx, tubal occlusion, and hysteroscopic insertion of Essure are currently recommended therapies to lower the hydrosalpingeal fluid amount [169].

## 4. Conclusions

Both similarities and dissimilarities characterize inflammatory processes occurring during embryo implantation and pathological states. Their course and severity are tightly controlled by numerous mechanisms. Specific molecules involved in both types of processes are observed. Their lack of expression may lead to implantation failures, miscarriages, and pregnancy pathologies. Knowledge of these processes will allow for their proper control, and regulation will allow for their appropriate course, which will affect the quality of our reproductive health.

## Figures and Tables

**Table 1:** Secreted cytokines that mediate inflammation and their role in the implantation process.

Pregnancy	Secreted Factor	Role	Reference
Preimplantation	TNFα	Induction of IL-1β secretion.	[6,7]
	IL-1β	Promotion/propagation of decidualization and modulation of maternal NK cells, secretion of chemokines, and other factors required for implantation. Enhanced glycoprotein fucosylation. Regulation of the synthesis/secretion of trophoblastic matrix metalloproteinases MMP-2, MMP-3, and MMP-9 involved in trophoblast invasion.	[15,16,17,18,19,20,21]
Implantation	IL-1β	Promotion/propagation of decidualization and modulation of maternal NK cells, secretion of chemokines, and other factors required for implantation.	[15,18,20]
	IL-6	Stimulation of migration and trophoblast invasion.	[22,23]
	IL-8	Stimulation of migration and trophoblast invasion.	[24,25]
	TNFα	Protection of the maternal tissue against excessive trophoblast invasion through the mechanism based on trophoblastic cell apoptosis. Regulation of synthesis/secretion of trophoblastic matrix metalloproteinases MMP-2, MMP-3, and MMP-9 participating in trophoblast invasion.	[26,27,28,29]
	IFNγ	Protection of the maternal tissue against excessive trophoblast invasion through the mechanism based on trophoblastic cells apoptosis.	[9,29,30,31]

Abbreviations: tumor necrosis factor α (TNF-α); interleukin 1β (IL-1β); interleukin 6 (IL-6); interleukin 8 (IL-8); interferon gamma (IFNγ).

## Data Availability

Not applicable.
